# Intramyocardial Hemorrhage: An Enigma for Cardiac MRI?

**DOI:** 10.1155/2015/859073

**Published:** 2015-02-01

**Authors:** Camilla Calvieri, Gabriele Masselli, Riccardo Monti, Matteo Spreca, Gian Franco Gualdi, Francesco Fedele

**Affiliations:** ^1^Department of Cardiovascular, Respiratory, Nephrologic and Geriatric Sciences, La Sapienza University of Rome, Viale del Policlinico 155, 00161 Rome, Italy; ^2^Department of Radiology, La Sapienza University of Rome, Viale del Policlinico 155, 00161 Rome, Italy

## Abstract

Cardiovascular magnetic resonance (CMR) is a useful noninvasive technique for determining the presence of microvascular obstruction (MVO) and intramyocardial hemorrhage (IMH), frequently occurring in patients after reperfused myocardial infarction (MI). MVO, or the so-called no-reflow phenomenon, is associated with adverse ventricular remodeling and a poor prognosis during follow-up. Similarly, IMH is considered a severe damage after revascularization by percutaneous primary coronary intervention (PPCI) or fibrinolysis, which represents a worse prognosis. However, the pathophysiology of IMH is not fully understood and imaging modalities might help to better understand that phenomenon. While, during the past decade, several studies examined the distribution patterns of late gadolinium enhancement with different CMR sequences, the standardized CMR protocol for assessment of IMH is not yet well established. The aim of this review is to evaluate the available literature on this issue, with particular regard to CMR sequences. New techniques, such as positron emission tomography/magnetic resonance imaging (PET/MRI), could be useful tools to explore molecular mechanisms of the myocardial infarction healing process.

## 1. Introduction

**Figure 1:**
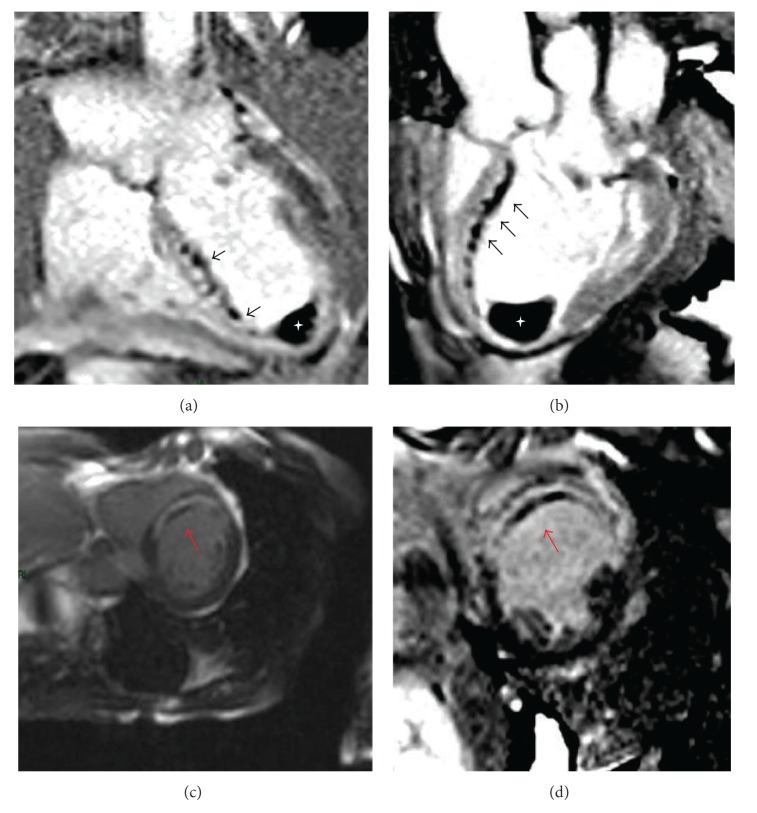
Microvascular obstruction on EGE and LGE imaging. Cardiovascular magnetic resonance has been performed 5 days after PCI in a 54-year-old male patient with anterior ST-elevation myocardial infarction. On T1 early gadolinium enhancement (EGE) images ((a), (b)), the persistent microvascular damage appears dark (so-called “dark zones”) (*black arrows*) within late gadolinium enhancement area, representing the myocardial MVO on septal and anterior wall. White asterisk is the massive thrombus inside the ventricle apex ((a), (b)). T1 LGE short axis images of the same patient ((c), (d)) show transmural late gadolinium enhancement on the anteroseptal wall and a dark zone within this area indicating MVO (*red arrows*).

“No Reflow” is a multifactorial phenomenon, also known as microvascular obstruction (MVO) ranging from 5% to 50%, occurring in patients having had a ST-elevation myocardial infarction (STEMI) after a primary percutaneous coronary intervention (PPCI) or thrombolysis. The MVO phenomenon is associated with a greater myocardial injury [1]. It has been defined as a patency restoration of an epicardial infarct-related coronary artery without complete microvascular reperfusion [1]. It has been described as a multifactorial pathogenesis including distal embolization, ischemia-reperfusion injury, and individual predisposition of coronary microcirculation injury [2]. MVO is generally assessed after PPCI, with myocardial reperfusion indexes as thrombolysis in myocardial infarction (TIMI) flow grade and myocardial blush grade (MBG) in the cath-lab and the electrocardiographic ST-segment elevation resolution (STR) in the coronary care unit. However, it can be better quantified by noninvasive imaging techniques, as cardiovascular magnetic resonance (CMR) [3]. Microvascular obstruction is associated with a worse prognosis at short and long time. In fact, it is a prognostic index of postinfarct adverse left ventricular (LV) remodeling, LV dysfunction, and increased mortality [4]. Microvascular obstruction is currently detected as hypoenhancement within bright regions of late gadolinium enhancement (LGE) on delayed postcontrast sequences, which are T1-weighted inversion recovery sequences acquired with different times after intravenous administration of gadolinium (Figure 1). Reperfusion injury may also cause intramyocardial hemorrhage (IMH) by erythrocytes extravasation through severely damaged endothelial walls [5]. IMH can be found in almost half of patients with successfully revascularized acute myocardial infarction (MI) and is associated with larger infarcts, presence of MVO, higher left ventricular volumes, lower ejection fraction, and the lack of improvement at follow-up. Therefore, it has been shown that the presence of IMH is associated with adverse LV remodeling and poor prognosis [6, 7].

**Figure 2:**
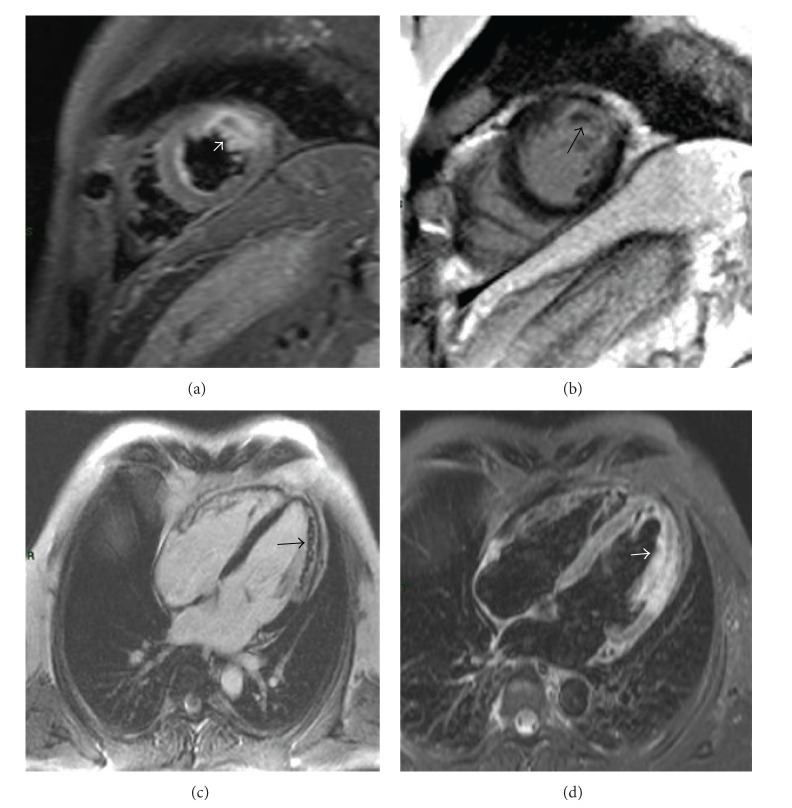
MVO and hemorrhagic areas in patient after reperfused anterolateral STEMI. ((a), (d)) In the anterolateral wall a large transmural hyperintense region and an hypointense core centrally located therein (*white arrows*) are present on the T2-short time inversion recovery (STIR) images in short axis and four chamber views showing edema on mid-apical anterolateral wall and an hypointense hemorrhagic area within the edema. ((b), (c)) Late gadolinium enhancement images show microvascular obstruction (*black arrows*), consistent with the hypointense area of haemorrhage, on mid-apical anterolateral wall.

IMH can be visualized by T2-weighted CMR sequences (Figure 2), because breakdown products of hemoglobin are paramagnetic and can influence magnetic properties of the tissue like the MR relaxation times (T1, T2, and T2^*^) [7, 8]. CMR is the only imaging modality able to exactly detect in vivo IMH.

Although IMH is present in a variable proportion of the patients after reperfused myocardial infarction and is closely related to infarct size, MVO and LV function, its prognostic significance still remains unclear.

Over the past decade, the rapid advances in noninvasive imaging techniques, such as myocardial contrast echocardiography (MCE) and in particular cardiovascular magnetic resonance, have enabled physicians to detect and quantify regions of MVO. In this review, we reported the available data about CMR sequences used to assess MVO and especially IMH.

## 2. No-Reflow Assessment: Cardiac MRI Prospective

A large amount of clinical data showed that MVO, detected by CMR, can predict early adverse LV remodeling, worse ejection fraction, larger myocardial infarct size, and major acute cardiac events (MACEs), independently from myocardial infarction size (IS) [9, 10].

Imaging techniques can provide a direct quantification of MVO extension and explain the relationship between IS and MVO in determining adverse LV remodeling. It is not yet well established what is the most accurate tool to visualize and quantify microvascular damage.

The evaluation of the molecular and cellular mechanisms after MI, during the first hours of ischemia-reperfusion, requires different types of sequences. In fact, microvascular obstruction is detected with different time-dependent sequences: first pass perfusion (FPP), early gadolinium enhancement (EGE) (Figure 1), and late gadolinium enhancement (LGE) (Figure 1) [11]. Previous studies demonstrated that the presence of MVO, detected on FPP images, has a prognostic role in STEMI patients [6] and its prevalence is higher compared with MVO assessed on LGE images [12]. Cochet et al. found MVO on FPP in 46% and MVO with LGE in 28% of patients with non-ST elevation myocardial infarction (NSTEMI). Thus, comparing Kaplan-Meier survival curves stratified by the presence of MVO on FPP and LGE sequences, they reported a strong relationship between MVO on LGE and MACEs (*P* < 0,001), suggesting its higher prognostic value in predicting cardiovascular events during follow-up [13].

Judd et al. conducted the first experimental study in 1995, in a reperfused canine infarct model, which showed correlation of CMR findings with pathology, by thioflavin-S staining [14]. The authors reported for the first time areas of no-reflow, the so-called “dark zones” characterized by hypoenhancement within bright regions of LGE. In humans, Lima et al. [15] described a well-defined time-intensity curve of different areas of enhancement occurring in human myocardial infarcts, based on the wash-in and wash-out kinetics of contrast agent.

Since 1998, as demonstrated by Wu et al. [9], MVO detected by delayed postcontrast CMR sequences became a strong predictor of adverse postinfarct LV remodeling. Then, this correlation was confirmed by studies of Hombach et al. [16] and Ørn et al. [17], which demonstrated that the presence of microvascular obstruction is associated with infarct size, transmural infarction, and increased volumes in both short term and long term follow-up, representing an index of severe myocardial damage and of adverse left ventricular remodeling. Recently, Hirsch et al. [18] compared no reflow on LGE images by CMR with early systolic retrograde flow on intracoronary flow measurements, showing a strong correlation between the two techniques. Angiographic blush indexes of myocardial reperfusion have been recently associated with the quantification of microvascular obstruction areas assessed by CMR [3].

Small studies demonstrated, using FFP sequences, a linear correlation between MVO areas detected by CMR and MBG severity assessed by PPCI [19, 20]. Several studies compared MVO (by either FFP or LGE sequences) with IS or transmural extent, showing conflicting results concerning their prognostic interest, with some reports favoring MVO and others favoring infarct size or transmurality as the best predictor of outcomes [9, 16, 21, 22]. Regarding this, it has recently been reported that the ratio between MVO and IS could be an index of irreversible damage, strongly influenced by time delays before PCI [23]. Nijveldt et al. [21] showed that late MVO is associated with increased wall thickness, regardless of the degree of infarct transmurality. Evidence strongly suggests that, in the acute setting, MVO might be more relevant than infarct size or transmural extent [22].

## 3. Intramyocardial Hemorrhage: An Enigma for Cardiovascular Magnetic Resonance?

IMH reflects severe reperfusion injury in acute myocardial infarction involving the structural and functional integrity of the microcirculation. Intramyocardial hemorrhage is frequently found in large reperfused myocardial infarctions and is strictly correlated to the presence of MVO (Figure 2) [12, 24]. Animal and human studies showed IMH prevalently within the infarct core of reperfused STEMI [24, 25]. In the prereperfusion era, intramyocardial hemorrhage was rarely seen on autopsy studies. Few studies in humans investigated the relationship of IMH with LV adverse remodeling and infarct size (Table 1). In the last decade, with the improvement of myocardial revascularization techniques, scientific attention to intramyocardial hemorrhage progressively increased. However, the clinical significance of IMH is still unclear, because of nonstandardized methods to detect IMH in vivo. Noninvasive techniques can provide further prognostic information and can evaluate the effect of preventive measures directed towards reducing reperfusion injury in the acute phase of MI. It is still unclear whether IMH could represent a marker of adverse LV remodeling beyond MI size, LV ejection fraction, and MVO.

As reported previously, IMH can be visualized by T2-weighted (T2W) sequences because hemorrhage and the breakdown products of oxygenated hemoglobin influence magnetic properties of the surrounding tissue [12]. T2W signal is high in the very early, hyperacute phase but then falls because of the paramagnetic effects of deoxyhemoglobin and intracellular methemoglobin, as demonstrated in cerebral hemorrhage [33]. Particularly in the core of the hematoma, where there is marked hypoxia, the signal may remain very low for a prolonged period of time [33, 34]. Instead, T2^*^-weighted CMR technique is very sensitive to the paramagnetic effects of deoxyhemoglobin and methemoglobin, but it requires relatively long echo times that may degrade image quality during cardiac imaging [11]. Finally, the standardized imaging method or protocol for assessment of IMH is still debated.

Asanuma et al. [32] described in humans an incidence of 38% of intramyocardial hemorrhage in patients with reperfused AMI. They demonstrated, in patients presenting with anterior AMI, the presence of IMH using myocardial contrast echocardiography (MCE) and T2^*^-weighted CMR sequences. Patients with IMH, compared to patients without IMH, showed a lower improvement of wall motion score, assessed by contrast echocardiography, at day 31 after coronary reflow. Ochiai et al. with the same CMR sequences reported an incidence of 33% of IMH and a strong correlation with infarct size, with less improvement of ejection fraction, using thallium-201 scintigraphy [31], confirming previous observations. Recently, the role of T2^*^-weighted sequences for detecting IMH was validated by histology in a study [35] which showed a strong correlation between hemorrhage sizes assessed in vivo with T2^*^ and assessed ex vivo by triphenyl-tetrazoliumchloride (TTC).

So far, the majority of studies, all prospective, used T2-weighted sequences to identify IMH, suggesting different points of view about its prognostic role.

Ganame et al. showed, in a multivariate analysis, that intramyocardial hemorrhage detected on T2-weighted images is an independent predictor of adverse LV remodeling at 4 months, regardless of infarct size [6]. Only two small studies [4, 7] did not show prognostic significance of hypointense cores beyond late MVO in prediction of functional changes at follow-up. The largest prospective study was conducted by Eitel and colleagues, which examined 346 STEMI patients. The authors demonstrated that the presence of IMH as a hypointense core in T2 weighted images in 35% of patients was associated with larger infarcts, greater amounts of microvascular obstruction, less myocardial salvage, and impaired left ventricular function. Of note, IMH, together with late MVO, was indicated as a strong predictor of MACEs at 6 months after MI [30]. For the first time the prognostic clinical relevance of the hypointense infarct cores in STEMI patients after reperfusion with PPCI has been demonstrated.

Other studies investigated the presence of IMH with T2-weighted and T2^*^ sequences suggesting a better definition of IMH with combined use of these two techniques.

Mather et al. [29] described an association between the presence of IMH, as a hypointense signal within the area at risk (AAR) on both T2W and T2^*^ images, and prolonged QRS duration, a marker of arrhythmic risk. Both were markers of adverse remodeling.

Recently, Kali et al. [28] compared the two types of sequences in humans and in dogs suggesting a superiority of T2^*^ for characterization of acute reperfusion myocardial hemorrhage. Moreover, using T2^*^ sequences, an iron deposit has been observed, measured by mass spectrometry within the areas of hemorrhagic infarction. This probably represents a prolonged inflammatory burden during the chronic phase of MI [27].

Previous studies [27–30, 35] identified intramyocardial hemorrhage as a hypointense signal or “negative contrast,” within a region of hyperintensity, by exploiting the T2 and T2^*^ shortening effects caused by the elevated myocardial densities of paramagnetic hemoglobin degradation products (deoxyhemoglobin, methemoglobin) or blood degradation products (ferritin and hemosiderin).

Pedersen et al. demonstrated for the first time a higher diagnostic sensitivity and specificity of T1-weighted inversion recovery sequences (T1WIR), compared to T2 short tau inversion recovery sequences (T2-STIR) and to T2^*^-weighted sequences. They were able to detect the presence of hemorrhage in porcine myocardium exposed to ischemia-reperfusion injury [36]. Moreover, a superior agreement of T1WIR sequences with pathology has been reported. Of note, this study showed that the combination of T1W and T2-STIR allows a better differentiation between MVO with and without IMH, by exploiting the T1 shortening effect of methemoglobin [36]. In fact, T1WIR sequences, depicting IMH as an area of hyperintense signal and normal myocardium as hypointense signal intensity, provide a superior image contrast.

Recently, Kandler et al., using T2^*^ mapping, found MVO on LGE images in 66% and IMH in 50% of patients after reperfused STEMI, which occurred in the majority of cases concomitantly with MVO [37] in the center of large MVO areas. These findings confirmed the strong interplay between occurrence of IMH and MVO [4] and the prevalence of IMH described in other studies [7]. Moreover, they compared patients with MVO and presence of IMH with ones without IMH, reporting a significantly lower EF, larger LV volumes (both systolic and diastolic), and larger infarct sizes in patients with IMH. In addition, concerning predictors of IMH presence, they revealed at multivariate analysis that MVO on LGE images and a “hypointense core” on T2 weighted images stated a high risk for the occurrence of IMH. Of note, this study demonstrated that defects in T2^*^ were also present in T2 imaging but not vice versa, suggesting that T2^*^ sequences are more accurate for IMH detection than T2 ones, probably for the signal intensity changes in the earlier stage of infarction. However, the optimal CMR sequences to assess IMH are still being debated.

## 4. Emerging Techniques: From Morphology to Function in Myocardial Infarction

Positron emission tomography/magnetic resonance imaging (PET/MRI) offers the potential for a powerful “one-stop shop” combination of structural, functional, and molecular imaging technology that may be superior to that of MRI imaging alone. PET has been considered a gold standard for clinical evaluation of myocardial viability [27, 36] in chronic ischemic heart disease, because the metabolic tracer [18F]-deoxyglucose (FDG) can distinguish ischemically compromised but viable “hibernating myocardium” [38]. The healing of myocardial infarction is a complex inflammatory process, resolved over time, which involves different cell types like monocytes and macrophages [39].

Simultaneous PET/MRI is an emerging technique combining two powerful imaging modalities [40]. In fact, PET/MRI is very attractive because it combines the high spatial resolution of contrast of magnetic resonance imaging in absence of ionizing radiation, with the high sensitivity of positron emission tomography (PET).

In fact, using 18F-fluorodeoxyglucose (18F-FDG) PET/MRI system, Lee et al. [41] examined myocardial inflammation postinfarction in mice with coronary ligation. They demonstrated a high 18F-FDG uptake on day 5 after myocardial infarction and that the number of monocytes/macrophages in the noninfarcted myocardium was lower than in the ischemic tissue.

Of note, also Fluorine 19 (19F) MRI has attracted much interest because of its capacity of cell tracking in vivo, useful to understand the molecular mechanism underlying reperfused myocardium [42].

Flogel et al. validated this emerging technique, in a murine model of acute cardiac and cerebral ischemia [43]. They used PFCs, nanoemulsion of perfluorocarbons, as a “positive” contrast agent, to detect inflammation in the border zones of infarction. Moreover, they confirmed with histology a colocalization of rhodamine-labeled PFCs with circulating monocytes/macrophages.

Recently, 9F/1H MRI has been used to visualize spatiotemporal recruitment of monocytes in reperfused MI in animals [42]. More interestingly, Ye et al. correlated monocyte infiltration with the presence of IMH and MVO, indicating a greater monocyte infiltration in MI areas with severe ischemia-reperfusion injury, assessed with IMH [40]. In fact, monocyte/macrophage infiltration was significantly impaired in MVO areas defined by MRI, compared to animals with IMH but no MVO [42]. This situation was associated with a worse LV functional outcome compared to MI isolated with IMH. Post-MI functional outcome is determined not only by MI size but also by the rate and quality of infarct healing. Myocardial infarction healing process causes changes in architecture and tissue properties; the principal cell effectors are monocytes which remove cell debris from granulation tissue and promote angiogenesis. In this regard, 19F/1H MRI can be clinically translatable into an innovative noninvasive approach to identify target patients. Moreover, this technique could be used to monitor medical therapies to optimize MI healing process.

In the future, this new technique may be more useful than cardiac MRI alone to detect labeled cells in reperfused MI areas, because the iron oxide nanoparticles recognition with cardiac MRI can be disturbed by magnetic susceptibility (T2^*^) effects on gradient echo magnetic resonance (MR) images. Therefore, it would be also useful to better detect IMH in vivo. Future research to evaluate the most appropriate clinical application of PET/MRI considering diagnostic performance, technical feasibility, practicality, and cost/benefit ratio compared to established diagnostic techniques will therefore be required before PET/MRI routine use.

## 5. Conclusions

IMH is a frequent finding in reperfused STEMI and it is often associated with reduced ventricular function and myocardial damage. In fact, it is a prognostic index of MACEs during follow-up, so it could be useful for a better risk stratification of patients with STEMI. IMH can be evaluated only by CMR, but there is still a debate about the optimal CMR sequences to detect it. Further research and maybe other imaging techniques, such as PET/MRI, could be necessary to resolve this open issue.

## Figures and Tables

**Table 1:** 

Study	Type of study	*N*°	IMH (%)	Time after MI	MVO (mean %)	IMH assessment	Follow-up	End point
Kidambi A et al. [26] (2013)	Prospective	39	35	2 days	56	T2-weighted and T2^*^	90 days	Infarct contractility

Kali et al. [27] (2013)	Prospective	15	73	3 days		T2^*^	6 months	Scar tissue

Kali et al. [28] (2013)	Prospective	/	14	3 days		T2 STIR and T2^*^	Unclear	Hemorrhage

Porto et al. [20] (2007)	Prospective	52	23	4–7 days		T2 W	6 months	Infarct size myocardial salvage index MVO

Mather et al. [29] (2011)	Prospective	48	25	2 days	63	T2 W and T2^*^	3 months	LVEF, LVES QRS myocardial salvage infarct size

Eitel et al. [30] (2011)	Prospective	346	35	3 days	43	T2-weighted	6 months	MACE: death, reinfarction congestive heart failure

Bekkers et al. [4] (2010)	Prospective	90	43	5 ± 2 days	54	T2-weighted	103 ± 11 days	LV remodeling

Beek et al. [7] (2010)	Prospective	45	44	5.1 ± 2.1 days	60	T2-weighted	4 months	Ejection fraction

Ganame et al. [6] (2009)	Prospective	98	25	1 week	64	T2-weighted	4 months	LV Adverse remodeling

Ochiai et al. [31] (1999)	Prospective	39	33	5.7 days	66	T2^*^-weighted gradient-echo	1 month	LVEF infarct size

Asanuma et al. [32] (1997)	Prospective	24	38	6 days	33	T2^*^-weighted gradient-echo	31 days	Wall motion score
